# Prevention of Neural Tube Defects in Europe: A Public Health Failure

**DOI:** 10.3389/fped.2021.647038

**Published:** 2021-06-24

**Authors:** Joan K. Morris, Marie-Claude Addor, Elisa Ballardini, Ingeborg Barisic, Laia Barrachina-Bonet, Paula Braz, Clara Cavero-Carbonell, Elly Den Hond, Ester Garne, Miriam Gatt, Martin Haeusler, Babak Khoshnood, Nathalie Lelong, Agnieszka Kinsner-Ovaskainen, Sonja Kiuru-Kuhlefelt, Kari Klungsoyr, Anna Latos-Bielenska, Elizabeth Limb, Mary T O'Mahony, Isabelle Perthus, Anna Pierini, Judith Rankin, Anke Rissmann, Florence Rouget, Gerardine Sayers, Antonin Sipek, Sarah Stevens, David Tucker, Christine Verellen-Dumoulin, Hermien E. K. de Walle, Diana Wellesley, Wladimir Wertelecki, Eva Bermejo-Sanchez

**Affiliations:** ^1^Population Health Research Institute, St. George's, University of London, London, United Kingdom; ^2^Department of Woman-Mother-Child, University Hospital Center, Centre Hospitalier Universitaire Vaudois (CHUV), Lausanne, Switzerland; ^3^Indagine Sulle Malformazioni Congenite in Emilia-Romagna (IMER) Registry (Emilia Romagna Registry of Birth Defects) Neonatal Intensive Care Unit, Pediatric Section Department of Medical Sciences, University of Ferrara, Ferrara, Italy; ^4^Centre of Excellence for Reproductive and Regenerative Medicine, Children's Hospital Zagreb, Medical School University of Zagreb, Zagreb, Croatia; ^5^Rare Diseases Research Unit, Foundation for the Promotion of Health and Biomedical Research in the Valencian Region, Valencia, Spain; ^6^Epidemiology Department, National Institute of Health Doutor Ricardo Jorge, Lisboa, Portugal; ^7^Health Department, Provincial Institute of Hygiene, Antwerp, Belgium; ^8^Paediatric Department, Hospital Lillebaelt Kolding, Kolding, Denmark; ^9^Directorate for Health Information and Research, Pietà, Malta; ^10^Department of Obstetrics and Gynaecology, Medical University of Graz, Graz, Austria; ^11^Université de Paris, Center of Research in Epidemiology and StatisticS/CRESS/Obstetrical Perinatal and Pediatric Epidemiology Research Team (EPOPé), INSERM, INRA, Paris, France; ^12^European Commission, Joint Research Centre, Ispra, Italy; ^13^Finnish Institute for Health and Welfare Terveyden Ja Hyvinvoinnin Laitos (THL), Register of Congenital Malformations, Helsinki, Finland; ^14^Department of Global Public Health and Primary Care, University of Bergen, Bergen, Norway; ^15^Division of Mental and Physical Health, Norwegian Institute of Public Health, Bergen, Norway; ^16^Department of Medical Genetics, Poznan University of Medical Sciences, Poznan, Poland; ^17^Health Service Executive-South, Department of Public Health, St. Finbarr's Hospital, Cork, Ireland; ^18^Auvergne Registry of Congenital Anomalies (CEMC-Auvergne), Department of Clinical Genetics, Centre de Référence des Maladies Rares, CHU Estaing, Clermont-Ferrand, France; ^19^Institute of Clinical Physiology, National Research Council, Pisa, Italy; ^20^Population Health Sciences Institute, Newcastle University, Newcastle, United Kingdom; ^21^Malformation Monitoring Centre Saxony-Anhalt, Medical Faculty Otto-von-Guericke-University, Magdeburg, Germany; ^22^Brittany Registry of Congenital Anomalies, CHU Rennes, Univ Rennes, INSERM, EHESP, Irset (Institut de recherche en santé, environnement et travail) - UMR_S 1085, Rennes, France; ^23^Health Intelligence R&D Health Service Executive, Dublin, Ireland; ^24^Department of Medical Biology and Genetics, 1st Faculty of Medicine, General University Hospital, Charles University, Prague, Czechia; ^25^Public Health England, London, United Kingdom; ^26^Congenital Anomaly Register and Information Service for Wales, Public Health Wales Knowledge Directorate, Singleton Hospital, Swansea, United Kingdom; ^27^Centre de Génétique Humaine, Institut de Pathologie et de Génétique, Charleroi, Belgium; ^28^Department of Genetics, Eurocat Northern Netherlands, University of Groningen, University Medical Center Groningen, Groningen, Netherlands; ^29^Wessex Clinical Genetics Service, Princess Anne Hospital, Southampton, United Kingdom; ^30^OMNI-Net Ukraine Programs, Rivne, Ukraine; ^31^Spanish Collaborative Study of Congenital Malformations (ECEMC), Unidad de Investigación sobre Anomalías Congénitas, Institute of Rare Diseases Research (IIER), Instituto de Salud Carlos III, Madrid, Spain

**Keywords:** neural tube defects, prevention, folic acid, fortification, Europe

## Abstract

**Objective:** Thirty years ago it was demonstrated that folic acid taken before pregnancy and in early pregnancy reduced the risk of a neural tube defect (NTD). Despite Public Health Initiatives across Europe recommending that women take 0.4 mg folic acid before becoming pregnant and during the first trimester, the prevalence of NTD pregnancies has not materially decreased in the EU since 1998, in contrast to the dramatic fall observed in the USA. This study aimed to estimate the number of NTD pregnancies that would have been prevented if flour had been fortified with folic acid in Europe from 1998 as it had been in the USA.

**Design and Setting:** The number of NTD pregnancies from 1998 to 2017 that would have been prevented if folic acid fortification had been implemented in the 28 countries who were members of the European Union in 2019 was predicted was predicted using data on NTD prevalence from 35 EUROCAT congenital anomaly registries and literature searches for population serum folate levels and folic acid supplementation.

**Results:** From 1998 to 2017 an estimated 95,213 NTD pregnancies occurred amongst 104 million births in the 28 countries in the EU, a prevalence of 0.92 per 1,000 births. The median serum folate level in Europe over this time period was estimated to be 14.1 μg/L. There is a lack of information about women taking folic acid supplements before becoming pregnant and during the first trimester of pregnancy, with one meta-analysis indicating that around 25% of women did so. An estimated 14,600 NTD pregnancies may have been prevented if the European countries had implemented fortification at the level adopted by the USA in 1998 and 25% of women took folic acid supplements. An estimated 19,500 NTD pregnancies would have been prevented if no women took folic acid supplements.

**Conclusions:** This study suggests that failure to implement mandatory folic acid fortification in the 28 European countries has caused, and continues to cause, neural tube defects to occur in almost 1,000 pregnancies every year.

## Introduction

Thirty years ago (in 1991) the Medical Research Council Vitamin Study randomized controlled trial showed that taking 4 mg folic acid before conception reduced the risk of a pregnancy with spina bifida, anencephaly or encephalocele (neural tube defects) by an estimated 72% (1). Dietary modification alone is not likely to achieve a high enough daily intake of folate to provide a reasonable reduction in risk; taking folic acid supplements or eating food fortified with folic acid is necessary.

Mandatory fortification of flour with folic acid has been introduced in 78 countries starting with the United States of America (USA) in 1998, over 20 years ago (2, 3). Despite evidence that such fortification is effective (2, 4–10), mandatory folic acid fortification of flour has not been introduced in Europe. Women in Europe are currently advised to take 0.4 mg folic acid (in the form of tablets or capsules) before pregnancy and in the first trimester to reduce their risk of a neural tube defect (NTD) pregnancy (11). The prevalence of NTDs has not decreased in Europe, with an estimated prevalence of 0.91 per 1,000 births from 1991 to 2011 (12) in comparison to the prevalence in Canada where the prevalence of NTDs decreased from 1.58 per 1,000 births before fortification to 0.86 per 1,000 births after fortification (6) and in the USA where the prevalence of spina bifida and anencephaly reduced from 0.76 per 1,000 births pre-fortification to 0.56 per 1,000 births after fortification (4).

Daly et al. demonstrated that there is a log linear association between a woman's serum folate level and her risk of having an NTD pregnancy (13). A meta-analysis by Wald et al. subsequently estimated that serum folate concentrations increase by 0.94 ng/mL for every 0.1 mg/day increase in folic acid intake in women aged 20–35 years (14). Wald combined these two results to predict the reduction in risk resulting from additional folic acid intake according to background serum folate concentration (14). This means that the risk reduction in NTD pregnancies resulting from the adoption of fortification can be predicted if the prevalence of NTDs in a country and the population serum folate levels in the country are known.

The aim of this study was to estimate the potential effect there would have been on the prevalence of NTDs over 20 years from 1998 to 2017 if fortification of flour with folic acid had been implemented in 1998 in Europe, as was done in the USA using data from European Congenital Anomaly Registries on the prevalence of NTDs and data from a literature search on population serum folate levels. An earlier study quantified the numbers of pregnancies with an NTD that could have been prevented in the UK and this study provides the relevant estimates for Europe.

## Methods

### Membership of Europe

In 2017 there were 28 countries belonging to the EU, 10 of which joined in 2004, two in 2007 and one in 2013. When estimating the numbers of NTDs that could be prevented in Europe from 1998, we have used two groups of countries—the 15 members in the EU in 1998 (EU15) and the 28 who were members of the EU in 2019 (EU28). In addition, we provide information on Norway, Switzerland and Ukraine, who are not members of the EU, but who have congenital anomaly registries that are members of EUROCAT (the European network of Congenital Anomaly Registries).

### Prevalence of Neural Tube Defect Pregnancies

EUROCAT currently surveys births from 39 registries in 21 countries in Europe (15, 16). The EUROCAT coverage varies over time according to which registries are able to provide data, with the maximum coverage being over 1.7 million births per year (39% of births in Europe). Details on the data and their collection are available at https://eu-rd-platform.jrc.ec.europa.eu/eurocat_en. Registries use multiple sources of information to ascertain cases including maternity, neonatal, and pediatric records; fetal medicine, cytogenetic, pathology, and medical genetics records; specialist services including pediatric cardiology; and hospital discharge and child health records. Anomalies were coded according to the International Classification of Disease (ICD) version 10 (17) (ICD-10) or ICD-9 with the British Pediatric Association (BPA) code extension for higher specificity and categorized by CA/syndrome subgroup (the system and the individual disorder), following the EUROCAT guidelines (18). NTDs include anencephalus, encephalocele and spina bifida. Cases were coded as having encephalocele only if there was no mention of anencephalus. Similarly, cases were classified as having spina bifida only if there was no mention of anencephalus or encephalocele. All cases with a known genetic condition were excluded.

Data about cases with an NTD [live births, late fetal deaths (≥20 weeks' gestation) and terminations of pregnancy for fetal anomaly at any gestation (TOPFA)] from 1st January 1998 to 31st December 2017 were obtained from the EUROCAT website (https://eu-rd-platform.jrc.ec.europa.eu/eurocat/eurocat-data/prevalence), as this website allows bespoke prevalence tables to be obtained interactively. Data were not available for all countries in Europe and for many countries the data available only covers births in particular regions and particular years (see Table 1). EUROCAT collects individual case data from full member registries, but only aggregate data from associate registries. Data from associate registries were only used if data were not available from a full registry in the same country. Table 1 shows which registries provided data to EUROCAT. Nine countries currently in the EU do not have any congenital anomaly registries that are members of EUROCAT. PubMed was searched for any additional information on the prevalence of NTDs in these countries from 1998 to 2017 using the terms “neural tube defect” and each individual country separately. Two studies were identified providing the prevalence for the Slovak Republic (20) and for Slovenia (21). For Luxembourg, the prevalence was assumed to be the same as that in Switzerland, for Greece and Cyprus the prevalence was assumed to be the same as that in Italy, for Estonia the prevalence was assumed to be the same as that in Finland, for Romania, the prevalence was assumed to be the same as that in Poland and for Latvia and Lithuania the prevalence was assumed to be the same as the average prevalence in Poland, Finland and Sweden.

**Table 1:** Number and prevalence of neural tube defect (NTD) pregnancies in EUROCAT registries reported from 1998 to 2017 and the proportion of the population covered over the whole 20 year period.

**Country**	**EU member**		**Registry**	**First year**	**Last year**	Total number of NTD pregnancies^*^	**Prevalence of NTD pregnancies per 1,000 births**	**Coverage of all 20 years national births data available^†^ (%)**
	**2017**	**1998**						
Belgium	Y	Y	Antwerp	1998	2016	271	0.73	15
Belgium	Y	Y	Hainaut	1998	2017	194	0.79	10
Denmark	Y	Y	Odense	1998	2015	105	1.14	7
France	Y	Y	Auvergne	2002	2015	170	1.05	1
France	Y	Y	Brittany	2011	2017	321	1.31	2
France	Y	Y	Paris	1998	2017	634	1.13	3
Italy	Y	Y	Emilia Romagna	1998	2017	391	0.57	6
Italy	Y	Y	Tuscany	1998	2017	317	0.56	5
Ireland	Y	Y	Cork and Kerry	1998	2017	210	1.16	14
Ireland	Y	Y	Dublin	1998	2012	255	0.70	28
Ireland	Y	Y	SE Ireland	1998	2016	137	1.05	10
Netherlands	Y	Y	North Netherlands	1998	2017	291	0.81	10
UK	Y	Y	East Midlands and South Yorkshire	1998	2017	1,288	1.13	8
UK	Y	Y	Northern England	2000	2017	775	1.35	4
UK	Y	Y	South West England	2005	2017	717	1.12	4
UK	Y	Y	Thames Valley	1998	2017	448	1.04	3
UK	Y	Y	Wales	1998	2017	942	1.41	4
UK	Y	Y	Wessex	1998	2017	684	1.20	4
Portugal	Y	Y	S. Portugal	1998	2017	156	0.43	18
Spain^‡^	Y	Y	Basque	1998	2016	374	1.02	4
Spain^‡^	Y	Y	Valencian Region	2007	2016	293	0.60	6
Germany	Y	Y	Mainz	1998	2014	89	1.63	0
Germany	Y	Y	Saxony-Anhalt	1998	2017	308	0.92	2
Austria	Y	Y	Styria	1998	2014	129	0.73	11
Finland	Y	Y	Finland ^(A)^	1998	2014	899	0.91	86
Sweden	Y	Y	Sweden ^(A)^	2007	2016	803	0.73	53
Croatia	Y	N	Zagreb	1998	2017	60	0.49	15
Malta	Y	N	Malta	1998	2016	73	0.92	96
Poland	Y	N	Wielkopolska	1999	2017	517	0.73	9
Bulgaria	Y	N	Sofia ^(A)^	1998	1999	40	2.07	1
Hungary	Y	N	Hungary ^(A)^	1998	2012	953	0.66	76
Czech Rep	Y	N	Czech Republic ^(A)^	2000	2010	848	0.74	55
Switzerland	N	N	Vaud	1998	2017	135	0.87	10
Norway	N	N	Norway	1999	2016	907	0.84	92
Ukraine	N	N	Ukraine	2005	2016	650	1.80	4

(A) *Associate registry*.

* *Includes NTDs occurring in live births, fetal deaths (stillbirths and later miscarriages ≥ 20 gestational age) and terminations of pregnancy for fetal anomaly excluding cases with known genetic conditions*.

† *This figure is the total number of births covered by the registry in the years reported divided by the total numbers of births occurring in the countries over the whole 20-year period*.

‡ *The prevalence is confirmed by the data from hospitals recording terminations of pregnancy for fetal anomalies in the Spanish Collaborative Study of Congenital Malformations (ECEMC)—an associate registry in EUROCAT which covers 16% of all births and includes hospitals from Valencian and Basque regions (19)*.

The annual prevalence of NTD pregnancies was estimated within each registry over the time period during which the registry was active. For countries in which there were several registries, the overall prevalence was estimated by calculating the total number of cases divided by the population births covered by the registries for each year. For European countries having registries located outside the geographical limits of Europe, only mainland registries were included (for example for France the registry on Isle de Reunion was not included). Previous studies have shown that the overall prevalence of NTD pregnancies in Europe has not significantly changed over this period (12, 22). Therefore, for years in which there was no registry reporting the prevalence, the average prevalence over the 3 previous years (where available) or the following 3 years (where available) was used.

### Population Serum Folate Levels

Any information from studies measuring serum folate levels in women of childbearing age was searched for in PubMed using the terms “folate” or “folic acid” and each individual European country separately. In addition, the above search was repeated using Google Scholar as this search engine also allows searching of country of authors. If several studies were available from a single country the largest study in the most recent years was used (see Table 2) (23–43). Serum folate has a log Gaussian distribution and therefore the median or geometric mean and centile values should be reported. Many studies presented the arithmetic mean and standard deviation (SD). The correct median and SD (on a log scale) were estimated by simulating different log Gaussian distributions and calculating the observed means and SDs until these calculated means and SDs matched the published values and then the median and SD (on a log scale) from the simulated distribution were used instead of the published values. If only the median was available and not the SD, the median SD from all the studies was used. As the same increase in folic acid will result in a greater risk reduction in women with lower serum folate compared with women with higher serum folate levels, we divided the serum folate levels in each country into quintiles and used the quintile medians to predict the risk reduction in each quintile of women separately. We then summed the quintiles to obtain the risk reduction in the whole population. We compared these estimates to the risk reduction using just the median folate level of the whole population. To determine how sensitive our estimates were to the reporting of serum folate levels in the different countries we also calculated the median serum folate level across all countries and used that value as the median serum folate level within each country.

**Table 2:** Estimated median folate levels and standard deviations (on a log scale) in European countries.

**Country**	**EU member**		**Median (nmol/L)**	**Standard deviation (log scale)**	**Source of information (reference)**
	**2017**	**1998**			
Austria	Y	Y	11.5	0.77	(23)
Belgium	Y	Y	13.8	0.41	(24)
Denmark	Y	Y	8.6	0.29	(25)
Finland	Y	Y	12.4	0.55	(26)
France	Y	Y	14.8	0.58	(27)
Germany	Y	Y	14.3	0.41	(28)
Greece	Y	Y	17.4	0.69	(29)
Ireland	Y	Y	16.2	0.53	(30)
Italy	Y	Y	10.4	0.45	(31)
Luxembourg	Y	Y			Values from Switzerland used
Netherlands	Y	Y	7.3	0.48	(32, 33)
Portugal	Y	Y	15.2	0.42	(34)
Spain	Y	Y	16.5	0.51	(35)
Sweden	Y	Y	15.0	0.40	(36)
United Kingdom	Y	Y	16.2	0.54	(30)
Bulgaria	Y	N			Values from Poland used
Croatia	Y	N	9.1		(37)
Cyprus	Y	N			Values from Greece used
Czech Republic	Y	N	14.0	0.38	(38)
Estonia	Y	N	12.4	0.60	(39)
Hungary	Y	N	19.0	0.45	(38)
Latvia	Y	N			Values from Poland used
Lithuania	Y	N			Values from Poland used
Malta	Y	N			Values from Greece used
Poland	Y	N	14.3	0.52	(40)
Romania	Y	N			Values from Poland used
Slovak Republic	Y	N	16.0	0.35	(41)
Slovenia	Y	N			Values from Poland used
Norway	N	N	7.3	0.50	(42)
Switzerland	N	N	14.3	0.67	(43)
Ukraine	N	N	[3.2]		Values estimated from prevalence of NTDs
Estimated median in countries in EU in 1998			14.1		Median weighted by numbers of births in each population.
Estimated median in countries in EU in 2019			14.1		Median weighted by numbers of births in each population.

### Folic Acid Supplementation

Recent studies have found that the proportion of women taking folic acid supplements at the correct time for prevention of NTDs (which is 4 weeks before pregnancy and the first 8 to 12 weeks of pregnancy) varies from around 10.4% (44, 45) up to 35% (46). An older meta-analysis of 34 studies found that around 25% of women took folic acid supplements periconceptually (47). Two estimates of the number of NTDs cases prevented were calculated; (i) Assuming that 25% of women took folic acid supplementation at the correct time and (ii) Ignoring any reductions in risk due to folic acid supplementation.

### Calculation of Number of Neural Tube Defects Prevented

In the USA, mandatory fortification with 140 μg of folic acid per 100 g of enriched cereal grain product was fully implemented in 1998 and has been estimated to provide 200 μg of folic acid per day to women of childbearing age (48). Wald et al. estimated that an increase in folic acid intake of 200 μg per day would increase the average serum folate concentration by 1.88 ng/ml or 4.26 nmol/L in women of childbearing age (14). We therefore estimated that the average serum folate level would have increased by 4.26 nmol/L in each country if fortification had occurred. The predicted reduction in prevalence if fortification had occurred is given by the formula: reduction in prevalence = (new serum folate/original serum folate)^−0.81^ from Wald et al. (14). For the scenario of 25% of women taking folic acid supplements, the effect of fortification is assumed to apply to only 75% of all births.

The total number of NTD diagnoses prevented from occurring annually in each country was estimated by multiplying the reduction in prevalence in the country by the total number of births in the country in that year. The numbers of births were obtained from Eurostat if they were not available from the EUROCAT registries (https://ec.europa.eu/eurostat/databrowser/view/TPS00204/default/table).

### Patient and Public Involvement

This study was not funded and therefore there were no funds or time allocated for PPI so we were unable to involve patients. We will invite patients to help us develop our dissemination strategy.

## Results

From 1998 to 2017 an estimated 95,213 NTD pregnancies occurred amongst 104 million births in the 28 countries in the EU in 2019, a prevalence of 0.92 per 1,000 births. The median serum folate level in Europe over this time period was estimated to be 14.1 nmol/L.

Table 3 shows that 14,600 NTD pregnancies could have been averted if the 28 countries in the EU in 2019 had implemented fortification at the level adopted by the USA in 1998, assuming that at least 25% of women in these countries had taken folic acid supplements before becoming pregnant and would therefore not have received a benefit from fortification. An estimated 19,500 NTD pregnancies could have been averted if it is assumed that no supplementation is occurring as then all women in the population are assumed to benefit from fortification. As it is likely that fewer than 25% of women in Europe did take supplements at the correct time and also that these women would have received some benefit from fortification the true estimate of the number of NTDs prevented lies between 14,600 and 19,500; a 15–21% reduction. This is equivalent to saying fortification could prevent almost 1,000 pregnancies with an NTD occurring every year in the 28 countries in the EU. If only those countries in the EU in 1998 had implemented fortification then the number of NTD pregnancies that would have been prevented lies between 12,000 and 16,000 (again 15% to 21% reduction).

**Table 3:** NTD: numbers of pregnancies and the prevalence if fortification had occurred from 1998 to 2017 according to European country.

**Country**	**EU member**			**Assuming no supplementation occurred**			**Assuming 25% of women took supplements (****47****)**		
	**2017**	**1998**	**Prevalence per** **1,000 births^†^**	**Prevalence per 1,000 births with fortification**	**Percentage reduction due to fortification (%)**	**Estimated NTD pregnancies prevented**	**Prevalence per 1,000 births with fortification**	**Percentage reduction due to fortification (%)**	**Estimated NTD pregnancies prevented**
Austria	Y	Y	0.71	0.53	25	279	0.57	19	209
Belgium	Y	Y	0.75	0.60	20	369	0.63	15	276
Denmark	Y	Y	1.12	0.80	28	394	0.88	21	296
Finland	Y	Y	0.90	0.70	23	234	0.75	17	175
France	Y	Y	1.16	0.93	20	3,735	0.99	15	2,801
Germany	Y	Y	1.02	0.82	20	2,893	0.87	15	2,170
Greece^b^	Y	Y	0.57	0.46	18	214	0.49	14	160
Ireland	Y	Y	0.93	0.75	18	222	0.80	14	166
Italy	Y	Y	0.57	0.43	25	1,526	0.46	19	1,145
Luxembourg^a^	Y	Y	0.87	0.70	20	20	0.74	15	15
Netherlands	Y	Y	0.82	0.55	32	962	0.62	24	721
Portugal	Y	Y	0.43	0.35	19	168	0.37	14	126
Spain	Y	Y	0.85	0.69	18	1,332	0.73	14	999
Sweden	Y	Y	0.79	0.64	19	315	0.67	14	237
United Kingdom	Y	Y	1.22	0.99	18	3,344	1.05	14	2,508
Bulgaria	Y	N	1.63	1.26	22	510	1.35	17	382
Croatia	Y	N	0.49	0.36	28	112	0.39	21	84
Cyprus^b^	Y	N	0.57	0.47	17	25	0.49	13	18
Czech Republic	Y	N	0.73	0.59	20	306	0.62	15	230
Estonia^c^	Y	N	0.90	0.70	23	58	0.75	17	43
Hungary	Y	N	0.64	0.54	16	193	0.56	12	145
Latvia^d^	Y	N	0.82	0.64	22	77	0.68	17	58
Lithuania^d^	Y	N	0.82	0.64	22	115	0.69	17	86
Malta	Y	N	0.92	0.76	17	13	0.80	13	10
Poland	Y	N	0.77	0.61	20	1,173	0.65	15	879
Romania^e^	Y	N	0.77	0.61	22	746	0.65	17	559
Slovak Republic^f^	Y	N	0.53	0.43	18	106	0.46	13	80
Slovenia^g^	Y	N	0.74	0.57	22	65	0.62	17	49
Norway	N	N	0.84	0.57	32	312	0.63	24	234
Switzerland	N	N	0.87	0.69	21	286	0.73	16	214
Ukraine	N	N	1.80	0.91	49	8,047	1.13	37	6,035
Countries in EU in 1998			0.95		21	16,007		15	12,005
Countries in EU in 2019			0.92		21	19,504		15	14,628

a *For Luxembourg the prevalence was assumed to be the same as that in Switzerland*.

b *For Greece and Cyprus the prevalence was assumed to be the same as that in Italy*.

c *For Estonia the prevalence was assumed to be the same as that in Finland*.

d *For Latvia and Lithuania the prevalence was assumed to be the same as the mean of Poland, Finland and Sweden*.

e *For Romania the prevalence was assumed to be the same as that in Poland*.

f *Prevalence in Slovak Republic from Behunova et al. (20)*.

g *Prevalence in Slovenia from Kokalj et al. (21)*.

† *The prevalence may differ slightly from that in Table 1 as in Table 3 it is the prevalence over the whole time period from 1998 to 2017 and in Table 1 it is the prevalence for the years of available data*.

The two different methods of estimating the effect of fortification used (that is using the median value or splitting the distribution into quintiles) gave similar answers to the nearest percentage point reduction in prevalence and therefore for simplicity the median method was used. Assuming that all countries had the same medium serum folate level (of 14.1 nmol/L) resulted in the reductions in NTD pregnancies being 15 and 20% (with and without supplementation, respectively). This would be expected as if the serum folate is assumed to be 10 nmol/L for example the predicted reduction in NTD pregnancies will be large, but if it is assumed to be 14.1 nmol/L the predicted reduction will be smaller. In other words, all those countries with serum folate below the median will have smaller reductions predicted if it is assumed their serum folate is the level of the median value. Similarly all those countries with serum folate levels above the median will have greater reductions predicted if it is assumed their serum folate is the level of the median. These effects are likely to be of a similar magnitude and hence the overall estimate remains similar.

## Discussion

This study suggests that failure to implement folic acid fortification has resulted in an estimated 15,000–20,000 pregnancies affected with an NTD in the EU from 1998 to 2017. In Chile, the level of flour fortification adopted was higher than that in the USA (2.2 μg/100 g flour vs. 1.4 μg/100 g). If this level of fortification had been adopted in the EU, the reduction in affected pregnancies would have been twice as large. Given the known benefits, there is no reason not to adopt the level of fortification introduced in Chile in all countries in the EU.

This paper has used robust methods to estimate the numbers of NTD-affected pregnancies that could have been prevented by fortification. The method was compared to an earlier study in the UK, which predicted that 2014 affected pregnancies could have been prevented in the UK from 1998 to 2012 (49). This compares with 2,508 in this study from 1998 to 2017 (given in Table 3) which is consistent with the earlier study.

A weakness of the study is that there is very little information on population serum folate levels in all the countries across the 20-year study period. Measurements of serum folate in women of childbearing age were obtained for 21 countries, but assumptions about serum folate needed to be made for the other countries (see Table 2). In addition, most serum folate values related to one point in time only and often from only a small sample of people as in Portugal with data only from a small sample of women and men. For Ukraine (not included in the European estimates but included in the study for completeness), there were no population estimates of serum folate. The prevalence of NTD pregnancies in Ukraine is extremely high and therefore this prevalence was used to estimate the serum folate levels—they would be expected to be considerably lower than any other European country. Serum folate levels were often analyzed in papers assuming serum folate had a Gaussian distribution, which is not a correct assumption and the median and SD (on a log scale) had to be estimated. However, the sensitivity analysis did demonstrate that assuming the folate levels were the same in all countries decreased the estimated numbers of NTDs prevented by only 1%, demonstrating the robustness of the estimation.

A further weakness in this study is that as there were no known NTD prevalence figures for several European countries, with assumptions needing to be made. These assumptions may not be correct. In addition, full member registries in EUROCAT submit individual case data which are extensively analyzed to ensure consistent coding within all EUROCAT registries. Associate member registries only submit aggregate data which limits the amount of data quality checks that can be performed on them. Even in those countries with EUROCAT registries, the registries may only cover a small proportion of all births in a country requiring generalization about the whole population to be made. For Spain the prevalence was obtained from two full member registries and confirmed by the prevalence in the associate registry—The Spanish Collaborative Study of Congenital Malformations (ECEMC) which has a greater coverage of 16% of births over the same time period (19).

Information on whether the mothers who had had an affected pregnancy took folic acid supplements before and during the first trimester of pregnancy would have been very informative, but unfortunately information on folic acid consumption is not well recorded in the EUROCAT registries, partly because such supplements are available as over the counter medications and do not require a prescription.

In many European countries preconceptual folic acid supplementation is not covered by the national health systems, which means that this important preventive measure is subject to individual's being able to afford it, which increases inequity in society.

The main obstacle for folic acid fortification was the concern that it may result in a proportion of the population consuming too much folate. In 2000, the European Commission Scientific Committee on Food report stated that “Although there is no conclusive evidence in humans, the Committee concludes that the risk of progression of the neurological symptoms in vitamin B12 deficient patients as a result of folic acid supplementation cannot be excluded and should be considered the most serious adverse effect” (50). However, a recent paper demonstrated that the evidence used by the Food and Nutrition Board at the Institute of Medicine (IOM) of the National Academies (formerly National Academy of Sciences) in the USA to develop the Dietary Reference Intake for folate was scientifically flawed and that there is no level of folate which increases the risk of progression of the neurological symptoms in vitamin B12 deficient patients (3).

This study suggests that the lack of adoption of an extremely cheap and effective intervention will continue to result in an estimated additional 1,000 affected pregnancies each year. We therefore suggest that all flour and other non-wheat products such as “gluten free” be fortified in the European countries with at least 2.2 μg Folic acid/100 g flour or equivalent.

## Data Availability Statement

All prevalence data analyzed in this study were obtained from the EUROCAT website (https://eu-rd-platform.jrc.ec.europa.eu/eurocat_en) and Table 2 provides all the data extracted from the referenced papers.

## Ethics Statement

Local procedures regarding ethics approval for the registries' activities and their collaborations with EUROCAT are available on the EUROCAT website (https://eu-rd-platform.jrc.ec.europa.eu/eurocat/eurocat-members/registries_en). The lead author affirms that this manuscript is an honest, accurate, and transparent account of the study being reported; that no important aspects of the study have been omitted; and that any discrepancies from the study as planned (and, if relevant, registered) have been explained.

## Author Contributions

JM: drafting/revising the manuscript for content, study concept or design, analysis of interpretation of data, acquisition of data, and statistical analysis. M-CA, EB, IB, LB-B, PB, CC-C, EDH, EG, MG, MH, BK, NL, AK-O, SK-K, KK, AL-B, EL, MO'M, IP, AP, JR, AR, FR, GS, AS, SS, DT, CV-D, HdW, DW, WW, and EB-S: drafting/revising the manuscript for content, acquisition of data. All authors contributed to the article and approved the submitted version.

## Conflict of Interest

The authors declare that the research was conducted in the absence of any commercial or financial relationships that could be construed as a potential conflict of interest.

## References

[1] MRC Vitamin Study Research Group. Prevention of neural tube defects: results of the Medical Research Council vitamin study. Lancet. (1991) 338:131–7. 10.1016/0140-6736(91)90133-A 1677062

[2] Honein MA Paulozzi LJ Mathews TJ Erickson JD Wong LY. Impact of folic acid fortification of the US food supply on the occurrence of neural tube defects. JAMA. (2001) 285:2981–6. 10.1001/jama.285.23.2981 11410096

[3] Wald NJ Morris J Blakemore C. Public health failure in the prevention of neural tube defects: time to abandon the Tolerable Upper Intake Level of folate. Public Health Rev. (2018) 39:2. 10.1186/s40985-018-0079-6 29450103 PMC5809909

[4] Williams LJ Mai CT Edmonds LD Shaw GM Kirby RS Hobbs CA. Prevalence of spina bifida and anencephaly during the transition to mandatory folic acid fortification in the United States. Teratology. (2002) 66:33–9. 10.1002/tera.10060 12115778

[5] Lopez-Camelo JS Orioli IM da Graca Dutra M Nazer-Herrera J Rivera N Ojeda ME. Reduction of birth prevalence rates of neural tube defects after folic acid fortification in Chile. Am J Med Genet A. (2005) 135:120–5. 10.1002/ajmg.a.30651 15846825

[6] De Wals P Tairou F Van Allen MI Uh SH Lowry RB Sibbald B. Reduction in neural-tube defects after folic acid fortification in Canada. N Engl J Med. (2007) 357:135–42. 10.1056/NEJMoa067103 17625125

[7] Yang QH Carter HK Mulinare J Berry RJ Friedman JM Erickson JD. Race-ethnicity differences in folic acid intake in women of childbearing age in the United States after folic acid fortification: findings from the National Health and Nutrition Examination Survey, 2001-2002. Am J Clin Nutr. (2007) 85:1409–16. 10.1093/ajcn/85.5.1409 17490980

[8] Sayed AR Bourne D Pattinson R Nixon J Henderson B. Decline in the prevalence of neural tube defects following folic acid fortification and its cost-benefit in South Africa. Birth Defects Res A Clin Mol Teratol. (2008) 82:211–6. 10.1002/bdra.20442 18338391

[9] Mathews T. Trends in Spina Bifida and Anencephalus in the United States, 1991-2006. Hyattsville, MD: NCHS Health E-Stat (2009).

[10] Collins JS Atkinson KK Dean JH Best RG Stevenson RE. Long term maintenance of neural tube defects prevention in a high prevalence state. J Pediatr. (2011) 159:143–9.e142. 10.1016/j.jpeds.2010.12.037 21345450 PMC3115395

[11] Cawley S Mullaney L McKeating A Farren M McCartney D Turner MJ. A review of European guidelines on periconceptional folic acid supplementation. Eur J Clin Nutr. (2016) 70:143–54. 10.1038/ejcn.2015.131 26350391

[12] Khoshnood B Loane M de Walle H Arriola L Addor MC Barisic I. Long term trends in prevalence of neural tube defects in Europe: population based study. BMJ. (2015) 351:h5949. 10.1136/bmj.h5949 26601850 PMC4658393

[13] Daly LE Kirke PN Molloy A Weir DG Scott JM. Folate levels and neural tube defects. Implications for prevention. JAMA. (1995) 274:1698–702. 10.1001/jama.1995.03530210052030 7474275

[14] Wald N Law M Morris J Wald D. Quantifying the effect of folic acid. Lancet. (2001) 358:2069–73. 10.1016/S0140-6736(01)07104-5 11755633

[15] Kinsner-Ovaskainen A Lanzoni M Garne E Loane M Morris J Neville A. A sustainable solution for the activities of the European network for surveillance of congenital anomalies: EUROCAT as part of the EU platform on rare diseases registration. Eur J Med Genet. (2018) 61:513–7. 10.1016/j.ejmg.2018.03.008 29597096

[16] Tucker FD Morris JK Neville A Garne E Kinsner-Ovaskainen A Lanzoni M. EUROCAT: an update on its functions and activities. J Commun Genet. (2018) 9:1–4. 10.1007/s12687-018-0367-3 PMC6167264 29736796

[17] World Health Organization. Congenital malformations, deformations and chromosomal abnormalities (Q00-Q99). In: International Statistical Classification of Diseases and Related Health Problems: 10th Revision. Geneva: World Health Organization (2010).

[18] EUROCAT. EUROCAT Guide 1.4 Online. Available online at: http://www.eurocat-network.eu/aboutus/datacollection/guidelinesforregistration/guide1_4 (accessed Feburary 2, 2018).

[19] Martínez-Frías ML. Postmarketing analysis of medicines: methodology and value of the spanish case-control study and surveillance system in preventing birth defects. Drug Saf. (2007) 30:307–16. 10.2165/00002018-200730040-00003 17408307

[20] Behunova J Klimcakova L Zavadilikova E Potocekova D Sykora P Podracka L. Methylenetetrahydrofolate reductase gene polymorphisms and neural tube defects epidemiology in the Slovak population. Birth Defects Res A Clin Mol Teratol. (2010) 88:695–700. 10.1002/bdra.20692 20672355

[21] Kokalj TS Rejc B Gersak K. Incidence and prevention of neural tube defects in Slovenia. Eur J Obstet Gynecol Reprod Biol. (2011) 156:119–20. 10.1016/j.ejogrb.2011.01.005 21300428

[22] Morris JK Springett AL Greenlees R Loane M Addor MC Arriola L. Trends in congenital anomalies in Europe from 1980 to 2012. PLoS ONE [Electronic Resource]. (2018) 13:e0194986. 10.1371/journal.pone.0194986 29621304 PMC5886482

[23] Majchrzak D Singer I Männer M Rust P Genser D Wagner KH. B-vitamin status and concentrations of homocysteine in Austrian omnivores, vegetarians and vegans. Ann Nutr Metab. (2006) 50:485–91. 10.1159/000095828 16988496

[24] De Laet C Wautrecht J-C Brasseur D Dramaix M Boeynaems J-M Decuyper J. Plasma homocysteine concentrations in a Belgian school-age population. Am J Clin Nutr. (1999) 69:968–72. 10.1093/ajcn/69.5.968 10232638

[25] Thuesen BH Husemoen LLN Ovesen L Jørgensen T Fenger M Linneberg A. Lifestyle and genetic determinants of folate and vitamin B12 levels in a general adult population. Br J Nutr. (2010) 103:1195–204. 10.1017/S0007114509992947 19968891

[26] Alfthan G Laurinen MS Valsta LM Pastinen T Aro A. Folate intake, plasma folate and homocysteine status in a random Finnish population. Eur J Clin Nutr. (2003) 57:81–8. 10.1038/sj.ejcn.1601507 12548301

[27] Chango A Potier De Courcy G Boisson F Guilland JC Barbe F Perrin MO. 5,10-methylenetetrahydrofolate reductase common mutations, folate status and plasma homocysteine in healthy French adults of the supplementation en vitamines et mineraux antioxydants (SU.VI.MAX) cohort. Br J Nutr. (2000) 84:891–6. 10.1017/S0007114500002518 11177206

[28] Fohr IP Prinz-Langenohl R Brönstrup A Bohlmann AM Nau H Berthold HK. 5,10-Methylenetetrahydrofolate reductase genotype determines the plasma homocysteine-lowering effect of supplementation with 5-methyltetrahydrofolate or folic acid in healthy young women. Am J Clin Nutr. (2002) 75:275–82. 10.1093/ajcn/75.2.275 11815318

[29] Hatzis CM Bertsias GK Linardakis M Scott JM Kafatos AG. Dietary and other lifestyle correlates of serum folate concentrations in a healthy adult population in Crete, Greece: a cross-sectional study. Nutr J. (2006) 5:5. 10.1186/1475-2891-5-5 16472386 PMC1431550

[30] Bates B Page P Cox L Nicholson S Roberts C Collions D. National Diet and Nutrition Survey Rolling Programme (NDNS RP). Supplementary Report: Blood Folate Results for the UK as a Whole, Scotland, Northern Ireland (Years 1 to 4 combined) and Wales (Years 2 to 5 combined). London: Public Health England (2017).

[31] Zappacosta B Persichilli S Iacoviello L Di Castelnuovo A Graziano M Gervasoni J. Folate, vitamin B12 and homocysteine status in an Italian blood donor population. Nutr Metab Cardiovasc Dis. (2013) 23:473–80. 10.1016/j.numecd.2011.10.001 22209740

[32] Melse-Boonstra A de Bree A Verhoef P Bjorke-Monsen AL Verschuren WM. Dietary monoglutamate and polyglutamate folate are associated with plasma folate concentrations in Dutch men and women aged 20-65 years. J Nutr. (2002) 132:1307–12. 10.1093/jn/132.6.1307 12042451

[33] De Bree A Verschuren WM Bjørke-Monsen A-L van der Put NM Heil SG Trijbels FJ. Effect of the methylenetetrahydrofolate reductase 677C → T mutation on the relations among folate intake and plasma folate and homocysteine concentrations in a general population sample. Am J Clin Nutr. (2003) 77:687–93. 10.1093/ajcn/77.3.687 12600862

[34] Castro R Rivera I Ravasco P Jakobs C Blom HJ Camilo ME. 5,10-methylenetetrahydrofolate reductase 677C → T and 1298A → C mutations are genetic determinants of elevated homocysteine. QJM Int J Med. (2003) 96:297–303. 10.1093/qjmed/hcg039 12651974

[35] Planells E Sánchez C Montellano MA Mataix J Llopis J. Vitamins B6 and B12 and folate status in an adult Mediterranean population. Eur J Clin Nutr. (2003) 57:777–85. 10.1038/sj.ejcn.1601610 12792662

[36] Van Guelpen B Hultdin J Johansson I Witthoft C Weinehall L Eliasson M. Plasma folate and total homocysteine levels are associated with the risk of myocardial infarction, independently of each other and of renal function. J Intern Med. (2009) 266:182–95. 10.1111/j.1365-2796.2009.02077.x 19298497

[37] Pucarin-Cvetkovic J Kaic-Rak A Matanic D Zah T Petrovic Z Car A. Dietary habits and folate status in women of childbearing age in Croatia. Coll Antropol. (2006) 30:97–102. 16617582

[38] Novaković R Cavelaars AEJM Bekkering GE Roman-Viñas B Ngo J Gurinović M. Micronutrient intake and status in Central and Eastern Europe compared with other European countries, results from the EURRECA network. Public Health Nutr. (2013) 16:824–40. 10.1017/S1368980012004077 22995736 PMC10271368

[39] Laanpere M Altmäe S Kaart T Stavreus-Evers A Nilsson TK Salumets A. Folate-metabolizing gene variants and pregnancy outcome of IVF. Reprod Biomed Online. (2011) 22:603–14. 10.1016/j.rbmo.2011.03.002 21507721

[40] Wartanowicz M Ziemlanski S Bulhak-Jachymczyk B Konopka L. Assessment of nutritional folate status and selected vitamin status of women of childbearing age. Eur J Clin Nutr. (2001) 55:743–7. 10.1038/sj.ejcn.1601217 11528487

[41] Krajčovičová-Kudláčková M Valachovičová M BlaŽíček P. Seasonal folate serum concentrations at different nutrition. Central Eur J Public Health. (2013) 21:36–8. 10.21101/cejph.a3785 23741898

[42] Vogiatzoglou A Smith AD Nurk E Berstad P Drevon CA Ueland PM. Dietary sources of vitamin B-12 and their association with plasma vitamin B-12 concentrations in the general population: the Hordaland Homocysteine Study. Am J Clin Nutr. (2009) 89:1078–87. 10.3945/ajcn.2008.26598 19190073

[43] Schupbach R Wegmuller R Berguerand C Bui M Herter-Aeberli I. Micronutrient status and intake in omnivores, vegetarians and vegans in Switzerland. Eur J Nutr. (2017) 56:283–93. 10.1007/s00394-015-1079-7 26502280

[44] Friberg AK. Few Danish pregnant women follow guidelines on periconceptional use of folic acid. Danish Med J. (2015) 62:A5019. 25748861

[45] Cawley S McCartney D Woodside JV Sweeney MR McDonnell R Molloy AM. Optimization of folic acid supplementation in the prevention of neural tube defects. J Public Health (Oxf). (2018) 40:827–34. 10.1093/pubmed/fdx137 29059388

[46] Bestwick JP Huttly WJ Morris JK Wald NJ. Prevention of neural tube defects: a cross-sectional study of the uptake of folic acid supplementation in nearly half a million women. PLoS ONE. (2014) 9:e89354. 10.1371/journal.pone.0089354 24586711 PMC3929694

[47] Ray JG Singh G Burrows RF. Evidence for suboptimal use of periconceptional folic acid supplements globally. Bjog. (2004) 111:399–408. 10.1111/j.1471-0528.2004.00115.x 15104602

[48] Rader JI Weaver CM Angyal G. Total folate in enriched cereal-grain products in the United States following fortification. Food Chem. (2000) 70:275–89. 10.1016/S0308-8146(00)00116-316093404

[49] Morris JK Rankin J Draper ES Kurinczuk JJ Springett A Tucker D. Prevention of neural tube defects in the UK: a missed opportunity. Arch Dis Child. (2016) 101:604–7. 10.1136/archdischild-2015-309226 26681697 PMC4941168

[50] ECSCF European Commission Scientific Committee on Food. Opinion on the Tolerable Upper Intake Level of Folate. Brussels: SCF/CS/NUT/UPPLEV/18 (2000).

